# Supplementary material for: ProteinShader: illustrative rendering of macromolecules
Source: BMC Struct Biol. 2009 Mar 30;9:19. doi: 10.1186/1472-6807-9-19 (PMC2672931; doi:10.1186/1472-6807-9-19)
Supplement: Additional file 1 — ProteinShader program without source code. This compressed file contains the complete ProteinShader program including associated libraries, but no source code. A README.txt file gives an overview of the ProteinShader distribution, and the index.html file in the help subdirectory has directions on getting started with the program as well as a set of tutorials. [file 1472-6807-9-19-S1.zip › ProteinShader-beta-0_9_4-binary/help/api/org/proteinshader/gui/components/controlpanel/package-frame.html]

org.proteinshader.gui.components.controlpanel (ProteinShader API)


org.proteinshader.gui.components.controlpanel

Classes 
  
AntialiasingPanel
  
AtomColorPanel
  
AtomScalePanel
  
AtomVisibilityPanel
  
CartoonColorPanel
  
CartoonSideChainsPanel
  
CartoonVisibilityPanel
  
ColorPanel
  
ControlPanel
  
DecorationsPanel
  
ModifierPanel
  
MotionPanel
  
OptimizePanel
  
RadioPanel
  
SelectorPanel
  
VisibilityPanel

Enums 
  
RadioPanel.Type
